# Optimized Whole Genome Association Scanning for Discovery of HLA Class I-Restricted Minor Histocompatibility Antigens

**DOI:** 10.3389/fimmu.2020.00659

**Published:** 2020-04-17

**Authors:** Kyra J. Fuchs, M. Willy Honders, Edith D. van der Meijden, Alwin E. Adriaans, Dyantha I. van der Lee, Margot J. Pont, Ramin Monajemi, Szymon M. Kielbasa, Peter A. C. ’t Hoen, Cornelis A. M. van Bergen, J. H. Frederik Falkenburg, Marieke Griffioen

**Affiliations:** ^1^Department of Hematology, Leiden University Medical Center, Leiden, Netherlands; ^2^Department of Internal Medicine, Hematology and Internal Oncology, University Hospital Erlangen, Erlangen, Germany; ^3^Immunotherapy Integrated Research Center, Fred Hutchinson Cancer Research Center, Seattle, WA, United States; ^4^Department of Biomedical Data Sciences, Leiden University Medical Center, Leiden, Netherlands; ^5^Department of Human Genetics, Leiden University Medical Center, Leiden, Netherlands; ^6^Centre for Molecular and Biomolecular Informatics, Radboud Institute for Molecular Life Sciences, Radboud University Medical Center, Nijmegen, Netherlands

**Keywords:** minor histocompatibility antigens, whole genome association scanning, allogeneic stem cell transplantation, HLA class I, graft versus host disease, Graft-versus-Leukemia effect, hematological diseases

## Abstract

Patients undergoing allogeneic stem cell transplantation as treatment for hematological diseases face the risk of Graft-versus-Host Disease as well as relapse. Graft-versus-Host Disease and the favorable Graft-versus-Leukemia effect are mediated by donor T cells recognizing polymorphic peptides, which are presented on the cell surface by HLA molecules and result from single nucleotide polymorphism alleles that are disparate between patient and donor. Identification of polymorphic HLA-binding peptides, designated minor histocompatibility antigens, has been a laborious procedure, and the number and scope for broad clinical use of these antigens therefore remain limited. Here, we present an optimized whole genome association approach for discovery of HLA class I minor histocompatibility antigens. T cell clones isolated from patients who responded to donor lymphocyte infusions after HLA-matched allogeneic stem cell transplantation were tested against a panel of 191 EBV-transformed B cells, which have been sequenced by the 1000 Genomes Project and selected for expression of seven common HLA class I alleles (HLA-A^∗^01:01, A^∗^02:01, A^∗^03:01, B^∗^07:02, B^∗^08:01, C^∗^07:01, and C^∗^07:02). By including all polymorphisms with minor allele frequencies above 0.01, we demonstrated that the new approach allows direct discovery of minor histocompatibility antigens as exemplified by seven new antigens in eight different HLA class I alleles including one antigen in HLA-A^∗^24:02 and HLA-A^∗^23:01, for which the method has not been originally designed. Our new whole genome association strategy is expected to rapidly augment the repertoire of HLA class I-restricted minor histocompatibility antigens that will become available for donor selection and clinical use to predict, follow or manipulate Graft-versus-Leukemia effect and Graft-versus-Host Disease after allogeneic stem cell transplantation.

## Introduction

Allogeneic stem cell transplantation (alloSCT) has a curative potential for treatment of hematological malignancies (1, 2). After alloSCT, however, patients still face the risk of disease relapse as well as Graft-versus-Host Disease (GvHD), both contributing to morbidity and mortality. A strategy to reduce GvHD is to deplete donor T cells from the stem cell graft followed by delayed administration of donor lymphocyte infusions (DLI) after alloSCT in order to mitigate relapse (3). Donor T cells that are present in the stem cell graft or DLI induce beneficial Graft-versus-Leukemia (GvL) reactivity as well as undesired GvHD by targeting polymorphic peptides, designated minor histocompatibility antigens (4–6).

Minor histocompatibility antigens are peptides produced by single nucleotide polymorphisms (SNPs), which differ between patient and donor, and are presented by HLA molecules on the cell surface (4–6). They are similar to neoantigens with respect to amino acid changes that are recognized by the immune system, but are encoded by germline polymorphisms instead of somatic mutations (7). This has the advantage that minor histocompatibility antigens are shared between patients independent of the disease. However, in contrast to somatic mutations, which are restricted to tumor cells or only a subclonal population, polymorphisms are present in all tissues. Therefore, the tissue distribution of minor histocompatibility antigens is a relevant factor for the type of clinical response that is induced after alloSCT. Donor T cells recognizing antigens that are broadly expressed on malignant cells and healthy tissues may induce GvL reactivity as well as GvHD, while donor T cells targeting antigens that are only expressed on (malignant) hematopoietic cells selectively mediate a GvL effect without GvHD.

Identification of minor histocompatibility antigens in GvL and GvHD is important to enable strategies to separate the two clinical effects (8). Characterizing antigens that induce GvHD may enable selection of a donor that is matched with the patient for the encoding SNPs. Moreover, in case a patient is transplanted with a donor mismatched for these SNPs, T cells for ubiquitous antigens may be selectively depleted from the stem cell graft or DLI. Hematopoietic-restricted minor histocompatibility antigens are relevant for therapeutic approaches aimed at enhancing immunity against the malignancy, either by vaccination (e.g., in the clinical trials 2012-002435-28, 2018-002752-33, NCT02528682) or adoptive T cell therapy (e.g., NCT03091933 and NCT03326921). Discovery of the dominant repertoire of minor histocompatibility antigens in GvL and GvHD that are often mismatched between patients and donors is therefore highly relevant for optimal development of strategies to separate GvL and GvHD after alloSCT.

**FIGURE 1:**
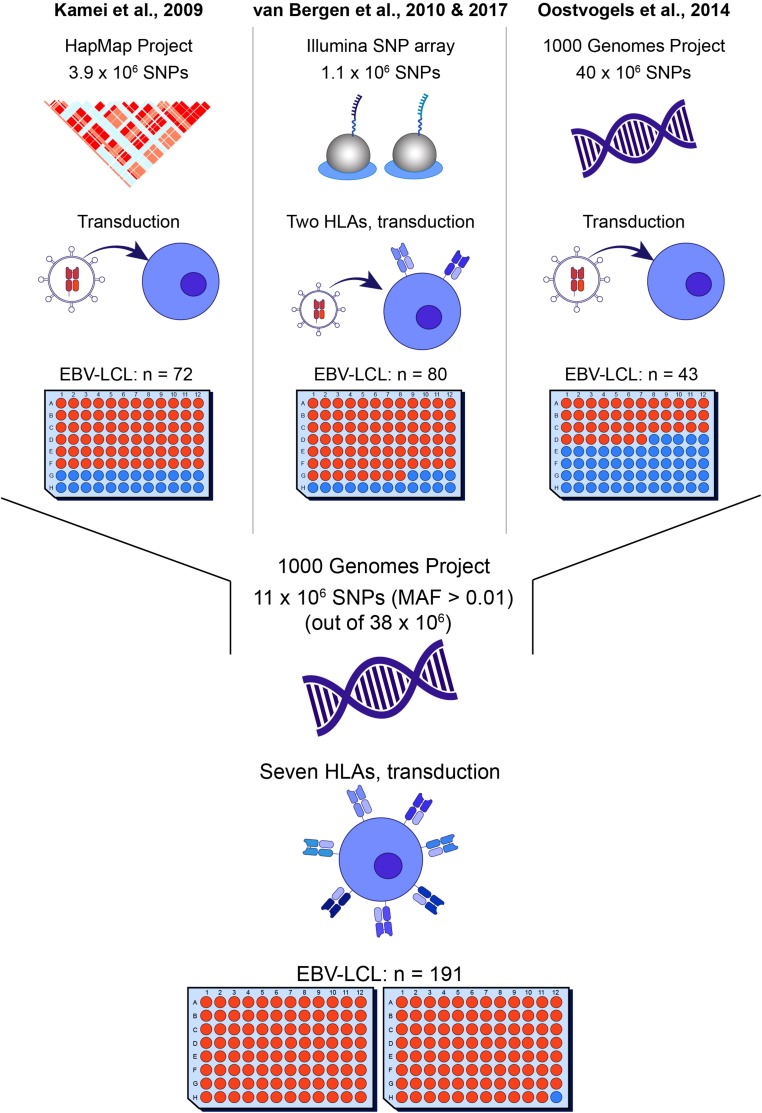
Overview of whole genome association methods for identification of minor histocompatibility antigens. Whole genome association scanning has been utilized for discovering minor histocompatibility antigens based on screening of SNPs of the HapMap Project [Kamei et al. (17)], measured by Illumina SNP array [van Bergen et al. (18)] and the 1000 Genomes Project [Oostvogels et al. (20)], using 43–80 EBV-LCLs, which had to be retrovirally transduced with the respective HLA restriction molecule or expressed only two HLAs (HLA-A*02:01 and B*07:02). The optimized panel allows screening for all SNPs and small indels of the 1000 Genomes Project and covers seven common HLAs (HLA-A*01:01, A*02:01, A*03:01, B*07:02, B*08:01, C*07:01, C*07:02) without the need for viral transduction of the HLA molecule with a panel size of 191 EBV-LCLs.

Currently, a total of 63 autosomal HLA class I minor histocompatibility antigens have been identified and confirmed as targets for T cell clones after alloSCT (5, 9–13). The first antigens were found by laborious methods, i.e., peptide elution (14), cDNA library screenings (15) and genetic linkage analyses (16). A significant improvement was achieved by introduction of whole genome association scanning (WGAs; Figure 1). Kamei et al. (17) tested T cell clones against a panel of Epstein-Barr virus-transformed lymphoblastoid cell lines (EBV-LCLs) and determined antigens by genetic mapping using the International HapMap Project. We refined the WGAs method by screening 1.1 million SNPs for association with recognition patterns of T cell clones against a panel of 80 EBV-LCLs, aimed at characterizing minor histocompatibility antigens presented by HLA-A^∗^02:01 and B^∗^07:02 (18). The SNPs for these EBV-LCLs were measured using a SNP-array and either directly encoded the antigen or served as markers in linkage disequilibrium with the antigen-encoding SNP that was not measured by the array. Using this approach, we discovered around 50% of the currently known HLA class I minor histocompatibility antigens (10–19).

In 2014, Oostvogels et al. (20) successfully identified an HLA class II minor histocompatibility antigen by scanning the genomes of 43 EBV-LCLs that were transduced with HLA-DPB1^∗^04:01 and sequenced as part of the 1000 Genomes Project. Here, we used EBV-LCLs that were sequenced as part of the 1000 Genomes Project to optimize the WGAs approach for the discovery of HLA class I minor histocompatibility antigens. A total of 191 EBV-LCLs were selected, allowing the screening of around 11 million SNPs and small indels (MAF > 0.01) in seven common HLA class I alleles (A^∗^01:01, A^∗^02:01, A^∗^03:01, B^∗^07:02, B^∗^08:01, C^∗^07:01, and C^∗^07:02). We explored the potential of the panel to identify antigens with different allele frequencies in each of the seven HLA class I alleles and, as proof of principle, tested the new panel using T cell clones recognizing known minor histocompatibility antigens. Furthermore, the panel was tested using T cell clones for unknown antigens. A total of seven new minor histocompatibility antigens were successfully identified in eight different HLA class I alleles. These peptides include one antigen in HLA-A^∗^23:01 and A^∗^24:02, for which the panel was not specifically designed, and one antigen, for which identification failed by our previous method by scanning 1.1 million SNPs and small indels, thereby confirming the improved efficiency of the WGAs approach to discover HLA class I minor histocompatibility antigens.

## Materials and Methods

All experiments have been performed according to standard biosecurity and institutional safety procedures.

### Patients

Peripheral blood and bone marrow samples were collected from six patients and their donors after approval by the LUMC Institutional Review Board (nos. P03.114, P03.173, and P04.003) and obtaining written informed consent according to the Declaration of Helsinki. The six patients underwent allogeneic stem cell transplantation for the treatment of acute myeloid leukemia (AML), chronic myeloid leukemia (CML) or myelodysplastic syndrome (MDS) and developed a clinical immune response after DLI characterized by GvHD or disappearance of patient cells in bone marrow or peripheral blood. Mononuclear cells were obtained by Ficoll-Isopaque gradient centrifugation and cryopreserved.

### T Cell Isolation and Culture

Peripheral blood mononuclear cells (PBMCs), which were obtained from patients at different time points after DLI during a detectable immune response, were thawed and enriched for T cells using an untouched pan-T cell isolation kit according to the manufacturer’s instructions (Miltenyi Biotec, Bergisch-Gladbach, Germany). Activated CD8^+^ T cells were either sorted based on expression of HLA-DR as *in vivo* activation marker or CD137 as *in vitro* activation marker. For *in vitro* activation, the enriched T cells were first stimulated with irradiated (15 Gy) patient PBMCs (E:T ratio of 1:2), which had been taken prior alloSCT, for 2 days in T cell medium [TCM; IMDM (Lonza, Walksersville, MD, United States), 5% fetal bovine serum (FBS; Bodinco, Alkmaar, Netherlands), 5% pooled human serum (Sanquin, Amsterdam, Netherlands), 1.5% glutamine (200 mM; Lonza, Walksersville, MD, United States), 1% penicillin/streptomycin (P/S; 200 mM; Lonza, Verviers, Belgium), 0.5 μg/ml amphotericin B (Bristol-Myers Squibb, Munich, Germany), 2 ng/ml IL-7 (Miltenyi Biotec), 2 ng/ml IL-15 (Miltenyi Biotec) supplemented with 20 IU/ml IL-2 (Novartis, Arnhem, Netherlands)]. Activated T cells were then stained with FITC-conjugated CD8 (clone RPA-T8, BD/Pharmingen, Breda, Netherlands) and APC-conjugated CD137 (clone MOPC-21, BD/Pharmingen) or HLA-DR (clone G46-6, BD/Pharmingen) and sorted on a BD FACS Aria device. Subsequently, T cells were dispensed at concentrations of 1, 3 or 10 cells/well in 384-well plates (Greiner Bio-One, Alphen a/d Rijn, Netherlands) and re-stimulated for expansion with irradiated (50 Gy) allogeneic PBMCs as feeders (25,000 cells/well) and 0.8 μg/ml phytohemagglutinin (PHA; Remel Europe, Dartford, United Kingdom) in TCM supplemented with 120 IU/ml IL-2. Re-stimulation was repeated on day 7 with 50,000 feeders/well and PHA. Growing T cell clones were afterward expanded by re-stimulation every 2 weeks with feeders at a ratio of 1:3-5. Experiments were performed on day 10–14 after re-stimulation.

### EBV-LCL Culture and Preparation

EBV-LCLs were generated from patient and donor PBMC or bone marrow samples using standard procedures and cultured in IMDM supplemented with 10% FBS, 1.5% glutamine and 1% P/S. A total of 191 EBV-LCLs from subjects sequenced as part of the 1000 Genomes Project were obtained from Coriell Cell Repositories, as part of the GEUVADIS project. In the GEUVADIS project, these EBV-LCLs have been analyzed by whole transcriptome RNA sequencing (21, 22). For the current project, cells were seeded in duplicate in 96-well plates at 60,000 cells/well. Plates were cryopreserved in multiple copies and used for WGAs as described below.

### T Cell Reactivity Assays

T cell clones were tested for recognition of EBV-LCLs by co-incubating 2,000 T cells with 15,000 EBV-LCLs (E:T ratio 1:7.5) loaded with or without peptide in TCM without IL-7 and IL-15 supplemented with 20 IU/ml IL-2 overnight in 384-well plates. Recognition of target cells was determined by measuring IFN-γ in the supernatant by ELISA according to manufacturer’s instructions (Sanquin, Amsterdam, Netherlands).

### SNP Data Files for Whole Genome Association Scanning

Data files containing biallelic SNPs and small indels aligned to GRCh37 for the 191 EBV-LCLs were downloaded from https://www.ebi.ac.uk/arrayexpress/experiments/E-GEUV-1/files/genotypes/. For 184 of the EBV-LCLs, genotyping had been retrieved from 1000 Genomes phase 1, while the remaining 17 samples had been imputed from Omni 2.5M SNP array data (21). As described by Lappalainen et al. (21), Gencode V12 had been used to functionally reannotate all variants and QTL mapping had been done with linear regression, using genetic variants with >5% frequency in 1-megabase window and normalized quantifications transformed to standard normal. Permutations had been used to adjust the false discovery rate to 5%. Using the software Plink 1.90 (23), data files were converted to binary files, merged and filtered for 191 selected EBV-LCLs and SNPs with a minor allele frequency above 0.01, resulting in a total number of 10,955,109 SNPs. Whole genome association scanning analysis.

T-cell clones exclusively recognizing patient-derived, but not donor EBV-LCLs were selected and tested for reactivity against 191 EBV-LCLs of the 1000 Genomes Project. The 191 EBV-LCLs were selected for expression of seven common HLA class I alleles, including HLA-A^∗^01:01, A^∗^02:01, A^∗^03:01, B^∗^07:02, B^∗^08:01, C^∗^07:01, and C^∗^07:02. HLA typing for individuals from the 1000 Genomes Project was obtained from Abi-Rached et al. (24). EBV-LCLs were also selected for co-expression of multiple of the seven HLA alleles to minimize the total number of cell lines required to compose a panel of EBV-LCLs aimed to include 50–100 cell lines per HLA. Since the panel is also used to determine HLA restriction of T cell clones, EBV-LCLs were as well specifically selected for expression of only one of two HLAs, which are often inherited in haplotype (e.g., B^∗^07:02 and C^∗^07:02; B^∗^08:01 and C^∗^07:01), in order to distinguish the molecules as separate HLA restriction alleles. The composition of the panel is shown in Supplementary Table S1. For each T-cell clone, EBV-LCLs were separated in positive and negative groups based on release of IFN-γ as measured by ELISA. HLA restriction of the T cell clones was determined by analyzing positive EBV-LCLs for shared expression of one of the HLA class I molecules as expressed by the patient and its donor. In a next step, positive and negative EBV-LCLs expressing the relevant HLA class I restriction allele were included in WGAs, while EBV-LCLs lacking the HLA allele were excluded. In WGAs, all 11 million SNPs were scanned for association with T cell recognition by means of the Fisher’s exact test using Plink 1.90 taking around half a minute per run. The Fisher’s exact calculates whether there is a significant difference in distribution of a SNP between EBV-LCLs that are recognized by the T cell versus EBV-LCLs that are not recognized by the T cell. Afterward, the reference SNP ID based on its chromosomal position and consequences of each SNP with a *p*-value cut-off at 10^–5^ were retrieved from Ensembl using biomaRt (25). SNPs that strongly associated with T cell recognition were further investigated. Coding sequences surrounding SNPs of interest were extracted from ensembl.org, translated into six reading frames and corresponding peptide sequences were searched for predicted HLA-binders using NetMHC 4.0 (26). Visualization of data was done using in-house scripts in R.

### Validation of New Minor Histocompatibility Antigens

Candidate peptides for minor histocompatibility antigens as well as their allelic variants were synthesized in house (purity >75%) and dissolved in DMSO. For validation, donor EBV-LCLs were pulsed with the peptides titrated in concentrations ranging from 50 μM to 1 pM and tested for recognition by the respective T cell clone by IFN-γ ELISA.

## Results

### Design of Optimized WGAs to Identify Minor Histocompatibility Antigens

In order to develop a more efficient WGAs method to identify minor histocompatibility antigens, 191 EBV-LCLs of the 1000 Genomes Project were selected for expression of seven common HLA class I alleles. These alleles include HLA-A^∗^01:01 (31.9%), A^∗^02:01 (49.9%), A^∗^03:01 (27.2%), B^∗^07:02 (27.9%), B^∗^08:01 (24.0%), C^∗^07:01 (28.9%), and C^∗^07:02 (30.8%) (percentages represent population frequencies calculated based on allele frequencies as reported in the Netherlands Leiden (*n* = 1305) population on www.allelefrequencies.net). In the finalized panel of 191 EBV-LCLs, each of the seven HLAs is expressed by 59–102 EBV-LCLs. In our selected panel of 191 EBV-LCLs, 176 cell lines are derived from individuals with Caucasian genetic background and 15 cell lines from individuals of Yoruba from Ibadan, Nigeria (Supplementary Figure S1A).

In order to evaluate the frequency of the selected HLAs in other human populations, we investigated HLA expression as reported for 2630 individuals in the five continental groups of the 1000 Genomes Project (24). In all human subpopulations, at least 43.5% of individuals express one or more of the seven selected HLAs (Supplementary Figure S1B), each expressed by at least 5% in each population with the exception of the East Asian group (Supplementary Figure S1C). In the European population, for which the panel has been designed, 87.2% of individuals express at least one of the selected HLAs and 56.9% of individuals express two or more common HLAs.

### *In silico* Evaluation of Optimized WGAs to Identify Minor Histocompatibility Antigens

**FIGURE 2:**
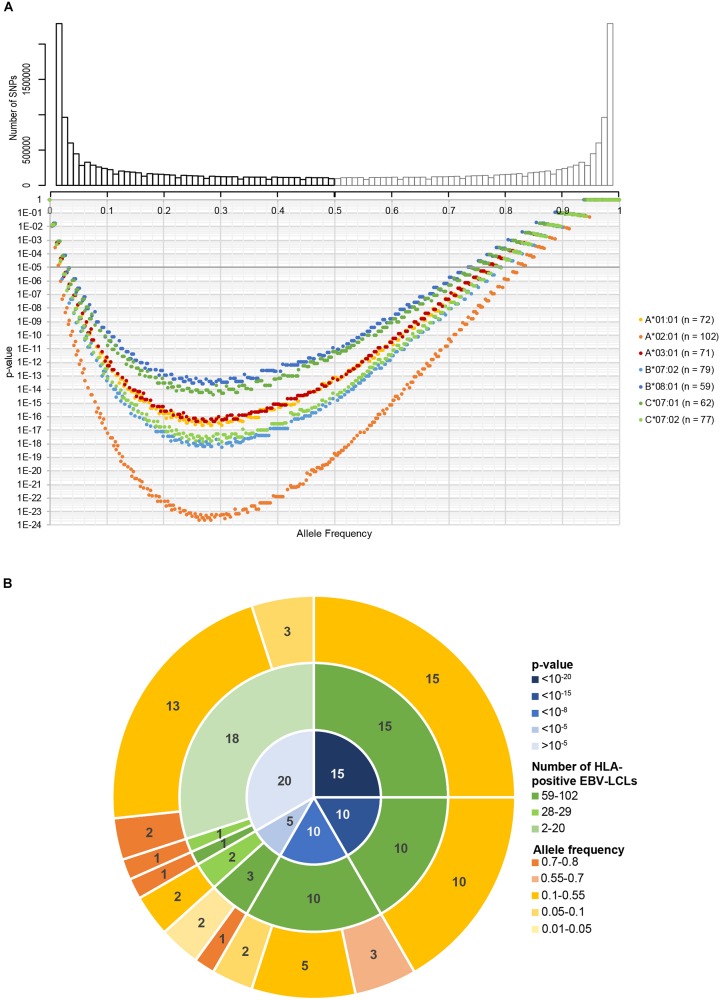
*In silico* evaluation of the optimized WGAs method to identify minor histocompatibility antigens. **(A)** In the optimized WGAs method, EBV-LCLs are scanned for 11 million SNPs with a minor allele frequency above 0.01. The upper graph shows the numbers of SNPs and their respective minor allele frequencies (black bars) in our panel of 191 EBV-LCLs from the 1000 Genomes Project. The major allele frequencies of the corresponding allelic variants (gray bars) are also shown. Based on the allele frequency and number of EBV-LCLs expressing HLA-A*01:01 (*n* = 72), A*02:01 (*n* = 102), A*03:01 (*n* = 71), B*07:01 (*n* = 79), B*08:01 (*n* = 59), C*07:01 (*n* = 62) and C*07:02 (*n* = 77), *p*-values were calculated using Fisher’s exact test. For the seven common HLA class I restriction alleles, the sample size of the panel is sufficient to identify SNPs with allele frequencies ranging between 0.02–0.82 (HLA-A*02:01) and 0.03–0.75 (HLA-B*08:01) with a *p*-value below 10^–5^. These allele frequencies correspond to population frequencies of 0.04–0.97 and 0.06–0.93, respectively, indicating that the majority of SNPs that are often mismatched in patient-donor pairs can be identified. **(B)** The panel of 191 EBV-LCLs from the 1000 Genomes Project was scanned for SNPs for 60 previously identified minor histocompatibility antigens that are presented by different HLA class I restriction alleles (Supplementary Table S2). Using Fisher’s exact test, *p*-values below 10^–5^ were calculated for 38 of the 39 antigens that are presented by one of the seven HLA class I alleles for which 59–102 EBV-LCLs are included in the panel. The only antigen with a *p*-value above 10^–5^ was HA-2 in HLA-A*02:01, which has a high population frequency of 0.99. In total, *p*-values below 10^–5^ were calculated for 40 of the 60 antigens, indicating that the optimized WGAs approach also allows identification of 2 antigens for which only 26–28 EBV-LCLs are included. Of the 20 antigens with *p*-values above 10^–5^, 19 antigens were presented by infrequent HLA alleles for which only 2–28 EBV-LCLs were included in the panel.

Using the 191 EBV-LCLs of the 1000 Genomes Project, we first performed an *in silico* analysis to evaluate the range of allele frequencies that can be identified for potential SNPs in seven common HLAs. For each HLA type, we predicted the number of EBV-LCLs that would be recognized (homo- or heterozygous for a specific SNP) or not (homozygous for its corresponding allelic variant). We then calculated the expected *p*-value for SNPs with different allele frequencies using the Fisher’s exact test. Allele frequencies between 0.03–0.73 and 0.02–0.82 corresponding to the HLA expressed by the lowest (HLA-B^∗^08:01, *n* = 59) and highest (HLA-A^∗^02:01, *n* = 102) number of EBV-LCLs result in *p*-values below our selected arbitrary *p*-value cut-off at 10^–5^ (Figure 2A). These allele frequencies correspond to population frequencies of 0.06–0.92 and 0.04–0.96, respectively, indicating that for the seven selected HLAs, the new WGAs approach should allow identification of the vast majority of minor histocompatibility antigens that are frequently mismatched in patient-donor pairs.

In a next step, we evaluated whether the panel is adequate to identify the SNPs for 60 known SNP-encoded HLA class-I minor histocompatibility antigens (Supplementary Table S2). For each antigen-encoding SNP, we calculated the *p*-value based on genotyping of all EBV-LCLs in our panel expressing the relevant HLA class I restriction allele (Figure 2B). In congruence with our prediction, our panel allows identification of 38 out of 39 SNPs encoding known antigens that are presented by one of seven common HLAs with a *p*-value below 10^–5^. Only one SNP that encodes HA-2 presented by HLA-A^∗^02:01, which has a high population frequency of 0.99, reaches a *p*-value above 10^–5^. In total, *p*-values below 10^–5^ were calculated for 40 antigens, indicating that two antigens could be identified that are presented by uncommon HLAs for which only 28–29 EBV-LCLs are included in the panel. These antigens are ACC-2D and ACC-1Y encoded by SNPs with allele frequencies of 0.24 and 0.25, resulting in *p*-values of 3.4 × 10^–7^ and 4.6 × 10^–6^ based on 28 (HLA-B^∗^44:02) and 29 (HLA-A^∗^24:02) EBV-LCLs in the panel, respectively. This implies that antigens presented by HLAs on a similar number of EBV-LCLs, namely, B^∗^35:01 (*n* = 29), C^∗^03:04 (*n* = 25), C^∗^04:01 (*n* = 47), C^∗^05:01 (*n* = 30) and C^∗^06:02 (*n* = 27) can be identified, provided that the allele frequency of the SNP is between 0.17–0.44 (*n* = 25) and 0.05–0.68 (*n* = 47) corresponding to a population frequency of 0.32–0.68 and 0.10–0.89, respectively, in order to reach a *p*-value below 10^–5^. The HLA class I alleles and corresponding range of allele frequencies for SNPs encoding minor histocompatibility antigens that can be directly identified by our optimized WGAs method are shown in Supplementary Table S3. ACC-2D is presented not only by HLA-B^∗^44:02, but also by HLA-B^∗^44:03, which increases the number of EBV-LCLs and lowers the *p*-value in the WGAs analysis to 8.2 × 10^–10^. Except for HA-2, the remaining 19 antigens, for which *p*-values above 10^–5^ were obtained, are presented by infrequent HLA alleles for which only 2–29 EBV-LCLs were included in the panel. In conclusion, the *in silico* analysis supported that our optimized WGAs approach allows direct identification of the majority of minor histocompatibility antigens in seven common HLA class I alleles.

### Performance of Optimized WGAs to Identify Known Minor Histocompatibility Antigens

**FIGURE 3:**
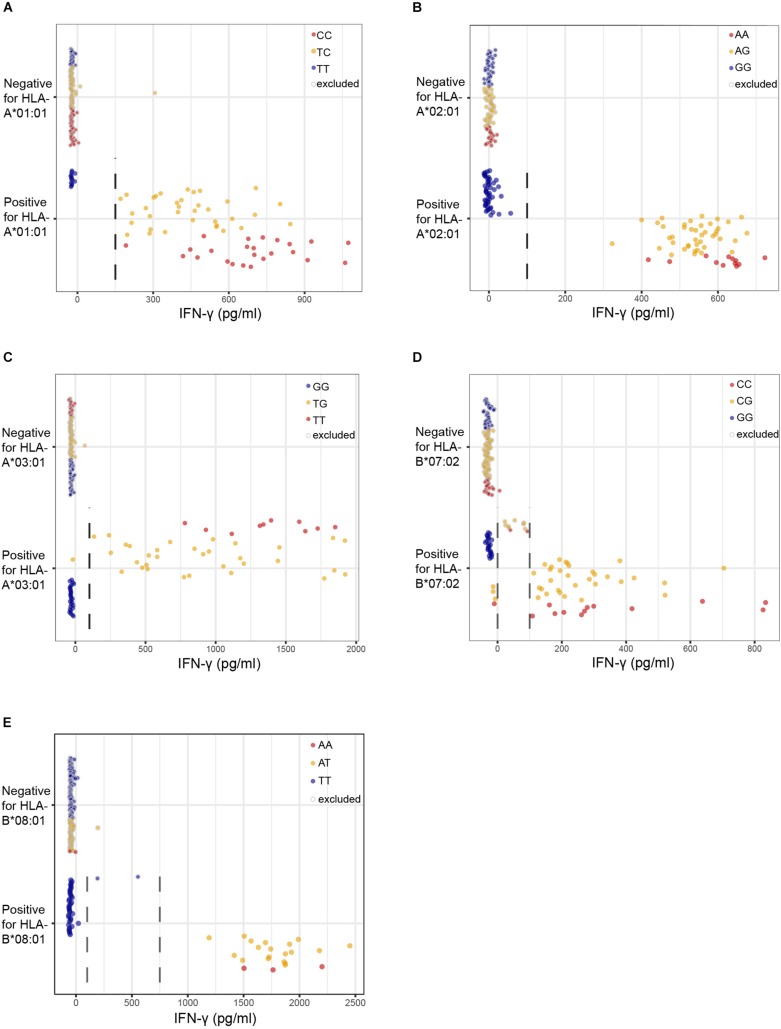
Performance of the optimized WGAs method to identify known minor histocompatibility antigens. T cell clones for HA-3T in HLA-A*01:01 **(A)**, HA-1 in A*02:01 **(B)**, LB-NADK-1K in A*03:01 **(C)**, LB-ARHGDIB-1R in B*07:02 **(D)** and LB-GEMIN4-1V in B*08:01 **(E)** were tested for reactivity against 191 EBV-LCLs from the 1000 Genomes Project by IFN-γ ELISA. EBV-LCLs are divided into groups based on presence or absence of the relevant HLA class I restriction allele. Dashed lines represent thresholds that were selected to divide the EBV-LCLs into negative or positive groups based on IFN-γ values for WGAs. EBV-LCLs with intermediate values, i.e., between two threshold lines, were excluded. EBV-LCLs that are homozygous or heterozygous for the antigen-encoding SNP are represented by red and orange dots, respectively. EBV-LCLs that are homozygous for the allelic variant are represented by blue dots. In WGAs, EBV-LCLs that are negative for the HLA restriction allele as well as EBV-LCLs with intermediate IFN-γ signals (gray border) were excluded from the analysis. The optimized WGAs approach correctly identified all antigen-encoding SNPs. The *p*-values as calculated by Fisher’s exact test are shown in Table 1 and Supplementary Table S4.

We first assessed the performance of our new panel of 191 EBV-LCLs in WGAs by testing reactivity of five T cell clones for known minor histocompatibility antigens in different HLA class I alleles (Table 1). Of the five antigen-encoding SNPs, two had been directly identified as associating SNPs by our previous WGAs method in which 80 EBV-LCLs were scanned for 1.1 million SNPs. The remaining three antigen-encoding SNPs were indirectly identified via associating SNPs that are inherited in haplotype. Since the 80 EBV-LCLs were selected for co-expression of HLA-A^∗^02:01 and B^∗^07:02, the other three HLA class I alleles (A^∗^01:01, A^∗^03:01, and B^∗^08:01) had to be retrovirally introduced. Here, we tested the five T cell clones for reactivity against the panel of 191 EBV-LCLs of the 1000 Genomes Project and measured release of IFN-γ by ELISA. EBV-LCLs expressing the relevant HLA restriction allele were divided into positive and negative groups based on T cell recognition. EBV-LCLs, which could not be assigned to either group due to intermediate IFN-γ signals as well as EBV-LCLs lacking expression of the relevant HLA restriction allele, were excluded from analysis. Using these T cell recognition patterns, all 11 million SNPs with a minor allele frequency above 0.01 were scanned for association using Plink 1.90. The SNPs that were identified with the new WGAs approach with *p*-values below 10^–5^ included all five antigen-encoding SNPs (Supplementary Table S4). Of the five SNPs, four were the strongest associating SNPs and one achieved second best *p*-value. Notably, comparing the genotypes of the antigen-encoding SNPs in EBV-LCLs with IFN-γ values measured by ELISA confirmed recognition patterns as expected for the T cell clones (Figure 3), and observed that statistical significance was only slightly less than predicted due to small numbers of excluded or incorrectly assigned EBV-LCLs (Table 1).

**TABLE 1:** Detection of known minor histocompatibility antigens by the optimized WGAs approach.

Patient	Clone	Antigen	SNP	Gene	HLA	AF^1^	Peptide^2^	Illumina 1M array^3^	Predicted *p*-value	Observed *p*-value
9528	H.9B8	HA-3T	rs2061821	*AKAP13*	A*01:01	0.58	V[**T**/M]EPGTAQY	y	4.15E-12	4.15E-12
7103	93-23	HA-1	rs1801284	*HMHA1*	A*02:01	0.32	VL[**H**/R]DDLLEA	y	5.41E-25	5.41E-25
8031	p29-001	LB-NADK-1K	rs4751	*NADK*	A*03:01	0.37	AVHNGLGE[**K**/N]GSQA	n	2.40E-16	3.41E-15
5852	10-144-10	LB-ARHGDIB-1R	rs4703	*ARHGDIB*	B*07:02	0.46	LPRACW[**R**/P]EA	n	2.59E-15	1.36E-10
5596	76-116	LB-GEMIN4-1V	rs4968104	*GEMIN4*	B*08:01	0.21	FPALRFVE[**V**/E]	n	5.40E-14	1.42E-13

*^1^Allele frequency in panel. ^2^Amino acid change that leads to antigen recognition is presented in brackets in bold. ^3^Part of Illumina Human 1M-duo SNP array.*

### Identification of New Minor Histocompatibility Antigens by the Optimized WGAs Approach

**FIGURE 4:**
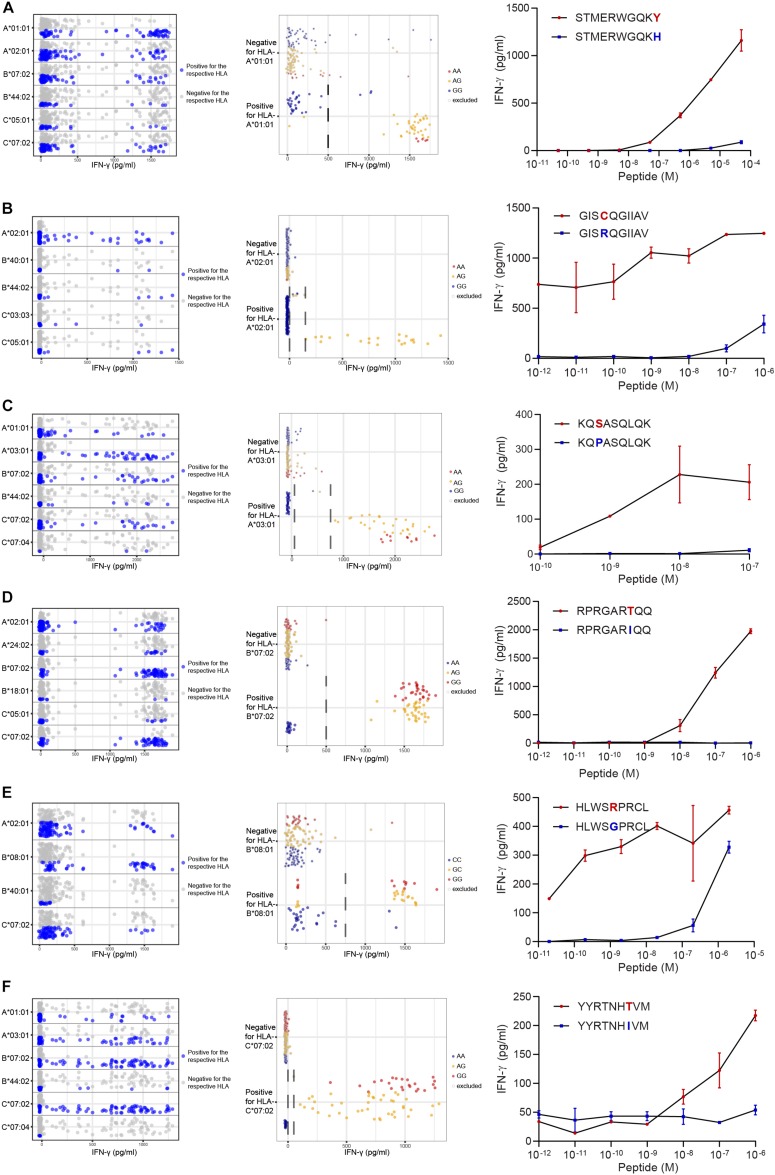
Identification of new minor histocompatibility antigens in common HLAs by optimized WGAs. T cell clones isolated from patients with hematological malignancies who responded to DLI after HLA-matched T-cell depleted alloSCT (Table 2), were tested for reactivity against 191 EBV-LCLs from the 1000 Genomes Project by IFN-γ ELISA. T-cell clones 14 **(A)**, 3B4 **(B)**, 2.1A12 **(C)**, 2–90 **(D)**, 4D8 **(E)** and H.9A6 **(F)** are shown. Data points show the IFN-γ release upon co-incubation with each of the 191 panel EBV-LCLs. First, HLA restriction was determined by analyzing EBV-LCLs that are recognized by the T cell clone for shared expression of one of the HLA class I alleles as expressed by the patient and donor (left graphs). For this purpose, the same dataset of IFN-γ values for 191 EBV-LCLs were separately displayed for each of the HLA class I alleles as expressed by the patient and donor. EBV-LCLs positive for the indicated HLA allele are shown by blue dots, while EBV-LCLs negative for this HLA are represented by gray dots. In a next step, EBV-LCLs are divided into positive and negative groups based on IFN-γ levels (indicated by dashed lines) while excluding those with intermediate IFN-γ levels (middle graphs) and WGAs is performed. SNPs that strongly associate with T cell recognition are analyzed for their distribution in EBV-LCLs that are positive and negative for the relevant SNP allele. EBV-LCLs that are homozygous or heterozygous for the associating SNP are represented by red and orange dots, respectively. EBV-LCLs that are homozygous for the allelic variant are indicated by blue dots. Gray borders represent EBV-LCLs that are excluded from the WGAs analysis based on intermediate IFN-γ levels or not expressing the relevant HLA allele. The middle graphs show results for rs4673 **(A)**, rs8069315 **(B)**, rs1050301 **(C)**, rs10749693 **(D)**, rs7080014 (rs7086691 not shown) **(E)** and rs1054487 **(F)**, which have been identified as associating SNPs by WGAs (Supplementary Table S5). The *p*-values of detection are shown in Table 2 and Supplementary Table S6. Finally, coding regions surrounding associating SNPs were searched for peptides with predicted binding to the respective HLA restriction allele by NetMHC 4.0. Peptide candidates for potential minor histocompatibility antigens and their allelic variants were synthesized, titrated and pulsed on donor EBV-LCLs and tested for T-cell recognition by IFN-γ ELISA (right graphs). Indicated are peptide sequences for LB-CYBA-1Y **(A)**, LB-DHX33-1C **(B)**, LB-IMMT-1S **(C)**, LB-YIPF1-1T **(D)**, LB-STK32C-1R **(E)** and LB-MAN2B1-1T **(F)**, which have been validated as minor histocompatibility antigens.

Finally, the performance of the optimized WGAs method was evaluated for seven T cell clones recognizing unknown minor histocompatibility antigens. All T cell clones were isolated from patients with hematological malignancies who developed immune responses in the form of GvHD or disappearance of patient hematopoietic cells after treatment with T-cell depleted alloSCT and DLI. PBMCs after DLI were enriched for CD3^+^ T cells and activated CD8^+^ T cells were isolated either by CD137 2 days after *in vitro* stimulation with patient hematopoietic cells obtained prior to alloSCT or directly using HLA-DR as *in vivo* activation marker. Growing T cell clones were selected based on reactivity against patient-derived EBV-LCLs, but not donor EBV-LCLs either unpulsed or pulsed with a peptide mix for the 63 known HLA class I minor histocompatibility antigens as analyzed above. Seven patient-specific T cell clones were selected and tested for reactivity against the panel of 191 EBV-LCLs to identify the minor histocompatibility antigens. For each T cell clone, EBV-LCLs were divided into positive and negative groups based on IFN-γ secretion (Supplementary Table S5). To determine HLA restriction of the T cell clones, positive EBV-LCLs were analyzed for shared expression of one of the HLA molecules as expressed by patient and donor (Figure 4). Subsequently, WGAs was performed as described above to identify SNPs that strongly associated with T cell recognition (Supplementary Table S6). Strongly associating SNPs identified by our optimized WGAs method were further investigated for encoding peptides with predicted HLA-binding using NetMHC 4.0. Peptide candidates as well as their allelic variants were subsequently pulsed on donor EBV-LCLs and tested for T cell recognition to validate minor histocompatibility antigens.

Following this strategy, we successfully identified minor histocompatibility antigens for seven T cell clones isolated from six patients (Table 2). Six of the seven new minor histocompatibility antigens are presented by HLA class I molecules for which the panel was designed. Of note, identification of LB-STK32C-1R as antigen recognized by HLA-B^∗^08:01-restricted T cell clone 4D8 failed with our previous WGAs method, but succeeded with the new approach. This antigen is encoded by two SNPs causing a single amino acid change. Both SNPs were not measured on the 1.1M Illumina SNP array, but are included in the 1000 Genomes Project data files.

**TABLE 2:** Novel minor histocompatibility antigens identified by the optimized WGAs approach.

Patient	Disease	Immune Response	Clone	Antigen	SNP	HLA	AF^1^	Peptide^2^	Type of variant	Illumina 1M array^3^	Observed *p*-value
2877	CML	Conversion	14	LB-CYBA-1Y	rs4673	A*01:01	0.34	STMERWGQK[**Y**/H]	missense variant	y	8.21E-11
7010	MDS	GvHD	3B4	LB-DHX33-1C	rs8069315	A*02:01	0.11	GIS[**C**/R]QGIIAV	missense variant	y	9.61E-17
9528	MDS	GvHD	2.1A12	LB-IMMT-1S	rs1050301	A*03:01	0.33	KQ[**S**/P]ASQLQK	missense variant	n	7.22E-18
5177	AML	GvHD	2-90	LB-YIPF1-1T	rs10749693	B*07:02	0.67	RPRGAR[**T**/I]QQ	5 prime UTR variant	n	6.83E-16
3087	CML	Conversion	4D8	LB-STK32C-1R	rs7080014, rs7086691	B*08:01	0.33	HLWS[**R**/G]PRCL	missense variant	n	2.67E-06
9528	MDS	GvHD	H.9A6	LB-MAN2B1-1T	rs1054487	C*07:02	0.33	YYRTNH[**T**/I]VM	missense variant	y	8.11E-11
6711	AML	GvHD	B1	LB-CYBA-2Y/LB-CYBA-3Y	rs4673	A*24:02/A*23:01	0.36	K[**Y**/H]MTAVVKLF	missense variant	y	7.95E-07

*^1^Allele frequency in panel. ^2^Amino acid change that leads to antigen recognition is presented in brackets in bold. ^3^Part of Illumina Human 1M-duo SNP array.*

**FIGURE 5:**
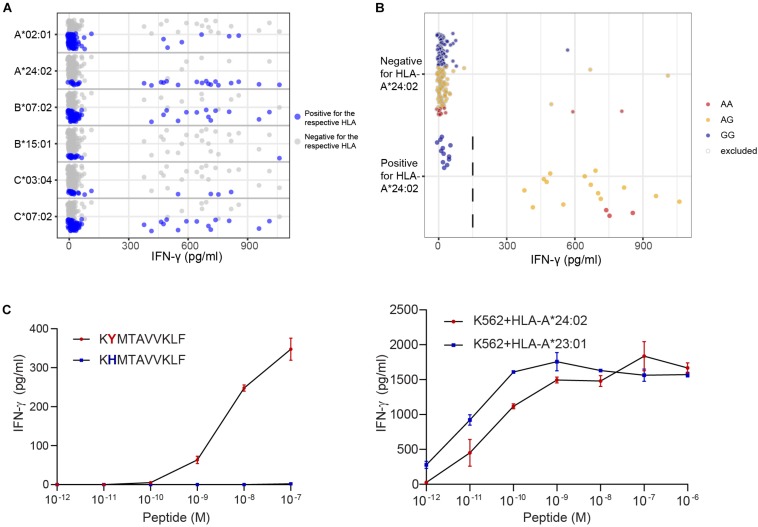
Identification of a novel minor histocompatibility antigen in uncommon HLAs. **(A)** Clone B1 from patient 6711 appeared to be restricted to HLA-A*24:02, which is expressed by 29 EBV-LCLs in our panel. **(B)** Due to an optimal allele frequency of 0.36, missense SNP rs4673 could be identified by WGAs. Of note, 4 of 6 EBV-LCLs that lacked expression of HLA-A*24:02 were positive for rs4673 and shared expression of HLA-A*23:01. **(C)** Analysis of the region surrounding rs4673 revealed a peptide with strong predicted binding to HLA-A*24:02 and A*23:01. Pulsing the peptide on donor EBV-LCLs (left) and K562 cells (right) transduced with HLA-A*24:02 or A*23:01 confirmed T-cell recognition of LB-CYBA-2Y in both HLA class I alleles.

Peptide STMERWGQKY has been identified as LB-CYBA-1Y in HLA-A^∗^01:01, whereas the same SNP also encodes peptide KYMTAVVKLF, identified as LB-CYBA-2Y. The latter peptide is presented by HLA-A^∗^24:02 (Figure 5A), for which our panel has not been specifically designed. Although HLA-A^∗^24:02 is expressed by only 29 EBV-LCLs of our panel, the SNP has an optimal allele frequency of 0.36, resulting in 17 EBV-LCLs that were recognized by the T cell clone and a *p*-value of 7.95 × 10^–7^ (Figure 5B). Another advantage of using a large panel of 191 EBV-LCLs is the possibility to identify antigens that are presented by more than one HLA restriction allele. For LB-CYBA-2Y, we noticed that six EBV-LCLs that lacked expression of HLA-A^∗^24:02 were recognized by the T cell clone. Four of these EBV-LCLs were positive for the SNP for LB-CYBA-2Y and shared expression of HLA-A^∗^23:01, an HLA molecule with a similar binding motif to HLA-A^∗^24:02. By pulsing the peptide on K562 cells transduced with either HLA-A^∗^24:02 or HLA-A^∗^23:01, LB-CYBA-2Y was validated as minor histocompatibility antigen presented by two HLA-A molecules (Figure 5C).

In conclusion, we have improved the WGAs method for identification of minor histocompatibility antigens by selecting a new concise 1000 Genomes Project EBV-LCL panel for seven common HLAs and demonstrated the value of this approach by successful discovery of seven novel minor histocompatibility antigens.

## Discussion

Identification of minor histocompatibility antigens is essential for improving the outcome of allogeneic stem cell transplantation. However, previous methods have been laborious and the number of identified antigens available for clinical use therefore remains limited. Here, we advanced current WGAs methods for discovery of minor histocompatibility antigens by designing a concise panel of 191 EBV-LCLs, which are sequenced as part of the 1000 Genomes Project, covering seven common HLA class I molecules.

For WGAs, CD8 T cell clones for unknown minor histocompatibility antigens were isolated from patients who had undergone alloSCT for treatment of hematological malignancies. These T cell clones were tested for reactivity against the new panel of 191 EBV-LCLs and subsequently screened for association with 10,955,109 biallelic SNPs and small indels with a minor allele frequency above 0.01. The genomic data do not contain gene deletions, but SNPs that are in strong linkage disequilibrium with common gene deletions (27) are included and can serve as markers for minor histocompatibility antigens encoded by these polymorphic genes, such as UGT2B17 (15, 17).

In our previous WGAs method, T cell reactivity was measured against a panel of 80 EBV-LCLs for which only 1.1 million SNPs were included on the Illumina SNP array (18). If the respective antigen-encoding SNP was not measured by the array, the antigen could only be indirectly identified via associating marker SNPs in linkage disequilibrium with the respective SNP. To evaluate the performance of our new panel of 191 EBV-LCLs, we selected T cell clones for five known minor histocompatibility antigens that are presented by different HLA class I alleles. Each of the five antigen-encoding SNPs was included in the list of strongly associating SNPs while only two SNPs were directly identifiable with the previous method. Likewise, for the seven novel minor histocompatibility antigens that were identified by the optimized WGAs approach, only 4 out of 8 SNPs were included on the 1.1 million Illumina SNP array.

The added value of the optimized WGAs strategy has also been confirmed by the discovery of LB-STK32C-1R, which is the target for an HLA-B^∗^08:01-restricted T cell clone, for which previous WGAs had failed. T cell clone 4D8, which recognized LB-STK32C-1R at peptide concentrations as low as 10^–11^ M, showed also reactivity against its allelic variant at concentrations >10^–8^ M (Figure 4E). Although clone 4D8 recognizes the allelic variant as exogenous peptide, it lacks reactivity against donor EBV-LCL, indicating that peptide presentation on the cell surface is not sufficient for T cell recognition when endogenously expressed. These data demonstrate that the TCR as expressed by clone 4D8 has a higher affinity for LB-STK32C-1R than for its allelic variant or, alternatively, that LB-STK32C-1R has a higher binding affinity for HLA-B^∗^08:01. The latter possibility is supported by NetMHC 4.0 showing that LB-STK32C-1R is predicted to bind strongly, while its allelic variant is predicted to bind weakly to HLA-B^∗^08:01. Furthermore, poor transportation by TAP, as reported for HA-8 (28) or proteasomal cleavage as demonstrated for HA-3 (29), may contribute to lack of recognition of the endogenous peptide.

Our optimized WGAs approach has been specifically designed to directly identify minor histocompatibility antigens in seven common HLAs without the need to retrovirally introduce these alleles for the European population. These common HLA molecules include HLA-A^∗^01:01, A^∗^02:01, A^∗^03:01, B^∗^07:02, B^∗^08:01, C^∗^07:01, and C^∗^07:02. In the European population, 87.2% of individuals express at least one and 56.9% express two or more of the seven HLA alleles. The identification of new minor histocompatibility antigens in six of these HLA molecules confirms the adequacy of the panel size and design of the approach. For the seven HLAs for which the method has been specifically designed, minor histocompatibility antigens can be directly identified for SNPs with a wide range of allele frequencies. In addition, minor histocompatibility antigens can be directly identified for a number of HLA alleles that are less common, but only for SNPs within a more restricted range of allele frequencies. Our *in silico* analysis showed that ACC-1Y in HLA-A^∗^24:02 (*n* = 29; number of EBV-LCLs expressing the HLA molecule) and ACC-1D in B^∗^44:02 (*n* = 28) could be identified and also one of the new minor histocompatibility antigen, i.e., LB-CYBA-2Y, was shown to be presented by HLA-A^∗^24:02. However, if the SNP has an allele frequency outside this restricted range, introduction of the HLA restriction allele into the panel of EBV-LCLs is necessary. Similarly, HLA alleles need to be introduced for all minor histocompatibility antigens that are presented by HLAs for which less than 25 EBV-LCLs are included in the panel. The HLA alleles and corresponding range of allele frequencies for SNPs encoding minor histocompatibility antigens that can be directly identified by our optimized WGAs method are shown in Supplementary Table S3. This table highlights the advantage of the new WGAs approach as compared to our previous method with 80 EBV-LCLs only expressing HLA-A^∗^02:01 and B^∗^07:02 and the method developed by Oostvogels et al. (20) using a panel of 43 EBV-LCLs from the 1000 Genomes Project in which the HLA restriction allele had to be introduced (Figure 1). Furthermore, the new WGAs method also allows for identification of antigens that are presented in more than one HLA as exemplified by LB-CYBA-2Y, which was found to be presented and recognized in HLA-A^∗^24:02 as well as A^∗^23:01. Presentation and recognition of the minor histocompatibility antigen in more than one HLA increases sample size, thereby enhancing the possibility to detect antigens in less frequent HLAs and expanding the range of SNPs with detectable allele frequencies.

As reported by Bykova et al. (30), SNPs with a high probability of mismatch between patient and donor have allele frequencies between 0.15 and 0.47. Our data showed that for seven common HLAs, all minor histocompatibility antigens encoded by SNPs with these allele frequencies can be detected with the new panel of EBV-LCLs from the 1000 Genomes Project. Only allele frequencies below 0.03 and above 0.73 corresponding to population frequencies below 0.06 and above 0.92, respectively, are outside of the predicted detection limits. The finding that all SNPs with a high probability of mismatch can be identified in seven common HLAs makes our optimized WGAs approach an ideal and more rapid strategy to identify the dominant repertoire of clinically relevant HLA class I-restricted minor histocompatibility antigens. Evidence that the repertoire of minor histocompatibility antigens is limited has been shown by Granados et al. (31) who predicted a maximum number of 50–100 antigens per HLA allele based on polymorphic peptides encoded by SNPs with a MAF of ≥0.05 that were identified for two HLAs on EBV-LCLs by mass spectrometry.

Due to high throughput sequencing techniques which enable genome wide detection of genetic variants, bioinformatic pipelines have been developed to predict neoantigens (32–35) and minor histocompatibility antigens (31, 36–40). Based on whole exome sequence data, Koparde et al. (39) found an average of 2463 non-synonymous SNP disparities in the Graft-versus-Host direction in patients transplanted with related donors, and an average of 4287 SNP disparities in patients transplanted with unrelated donors (39). SNP disparities in the same range have been reported by others (37). Martin et al. (40) showed that a higher number of SNP disparities in patients transplanted with sibling donors was associated with an increase in grade III-IV GVHD and stage 2–4 acute gut GVHD, whereas Ritari et al. (41) found an association between a higher number of mismatching peptide ligands and chronic GvHD. All SNP disparities as measured by Koparde et al. (39) were also investigated to encode 9-mer peptides with predicted binding to patients’ HLA class I alleles using NetMHCpan 2.8. The results revealed 3670 peptides with intermediate and 852 peptides with strong binding in patients transplanted with related donors, and 5386 intermediate and 1160 strong binding peptides in patients with unrelated donors. Although minor histocompatibility antigens are probably present among these peptides, prediction tools are hampered by high false discovery rates due to failure to accurately predict intracellular HLA class I peptide processing. Whole transcriptome RNA sequencing and HLA ligandome analysis by mass spectrometry can be implemented as additional steps to select for peptides that are expressed and presented on the cell surface. These techniques significantly decrease false discovery (31, 38, 41, 42), but also reduce the sensitivity and lead to a higher chance that antigens are missed (38), illustrating that prediction tools for minor histocompatibility antigens still require optimization.

Whole genome association scanning is a technique that allows discovery of minor histocompatibility antigens with high sensitivity and specificity. The method is rapid and cost effective, since one panel of 191 EBV-LCLs can be used to identify antigens for T cells from different patients. Another advantage is that discovery of minor histocompatibility antigens is not restricted to non-synonymous SNPs, but can also be performed for other polymorphisms, such as synonymous SNPs in alternative reading frames and intron SNPs in alternative splice variants. In order to perform WGAs, T cell clones are needed that are able to recognize EBV-LCLs. Since T cell responses after alloSCT may have been induced by professional antigen-presenting cells, there is a possibility that minor histocompatibility antigens exist that are myeloid specific which cannot be identified by our EBV-LCL panel. Furthermore, for antigens that are not encoded by SNPs such as neoantigens or Y chromosome encoded antigens, other techniques such as peptide (34) or mini-gene libraries (33) have to be employed.

Here, we optimized WGAs to enable discovery of the dominant repertoire of minor histocompatibility antigens in common HLA class I alleles. Discovery of this repertoire is relevant to predict and manipulate GvL and GvHD after alloSCT. Discovery of immunogenic antigens is also important to gain insight into the various cut-offs that need to be applied in prediction tools for minor histocompatibility antigens, which are particularly necessary to characterize antigens for HLA alleles and SNP mismatches that are rarer. As such, WGAs together with prediction tools may ultimately enable development of personalized strategies to separate GvL from GvHD, thereby improving clinical outcome after alloSCT.

## Data Availability Statement

Publicly available datasets were analyzed in this study. This data can be found here: https://www.ebi.ac.uk/arrayexpress/experiments/E-GEUV-1/files/genotypes/.

## Ethics Statement

The studies involving human participants were reviewed and approved by the LUMC Institutional Review Board. The patients/participants provided their written informed consent to participate in this study.

## Author Contributions

KF, MH, EM, JF, and MG designed the research. KF, MH, EM, AA, DL, and MP performed the experiments. KF, MH, EM, AA, DL, MP, CB, JF, and MG analyzed the results. KF, RM, SK, and P’tH contributed to the bioinformatic analysis. JF and MG supervised the research. KF, SK, P’tH, CB, JF, and MG wrote the manuscript. All authors read and reviewed the manuscript.

## Conflict of Interest

The authors declare that the research was conducted in the absence of any commercial or financial relationships that could be construed as a potential conflict of interest.

## Supplementary Material

FIGURE S1 Regional origin of selected EBV-LCLs and common HLA distribution in continental groups of the 1000 Genomes Project. **(A)** The 191 selected EBV-LCLs from the 1000 Genomes Project are of different regional origin, including 176 (92.1%) of European origin (CEU: Utah Residents with Northern and Western European Ancestry, FIN: Finnish in Finland, GBR: British in England and Scotland, TSI: Toscani in Itay) and 15 (7.9%) belonging to individuals from Yoruba in Ibadan, Nigeria (YRI). **(B)** HLA typing was obtained from 2693 individuals in the 1000 Genomes Project to determine the frequencies of the seven common HLA class I alleles in different human subpopulations. After exclusion of 63 individuals with unclear subtypes, the occurrence in individuals **(B)** and population frequency **(C)** of HLA-A^∗^01:01, A^∗^02:01, A^∗^03:01, B^∗^07:02, B^∗^08:01, C^∗^07:01, and C^∗^07:02 was counted for individuals of European (EUR, *n* = 521), admixed American (AMR, *n* = 363), South Asian (SAS, *n* = 522), African (AFR, *n* = 697) and East Asian (EAS, *n* = 527) background.

TABLE S1 HLA co-expression in the EBV-LCL panel.

TABLE S2 In silico analysis of published antigens.

TABLE S3 Detectable ranges of minor allele frequencies.

TABLE S4 SNP associations for clones recognizing known minor histocompatibility antigens.

TABLE S5 Genotyping for EBV-LCL panel of antigen-encoding SNPs.

TABLE S6 SNP association of new minor histocompatibility antigens.
