# Supplementary figures and images for: Optimized Whole Genome Association Scanning for Discovery of HLA Class I-Restricted Minor Histocompatibility Antigens
Source: Front Immunol. 2020 Apr 17;11:659. doi: 10.3389/fimmu.2020.00659 (PMC7180171; doi:10.3389/fimmu.2020.00659)

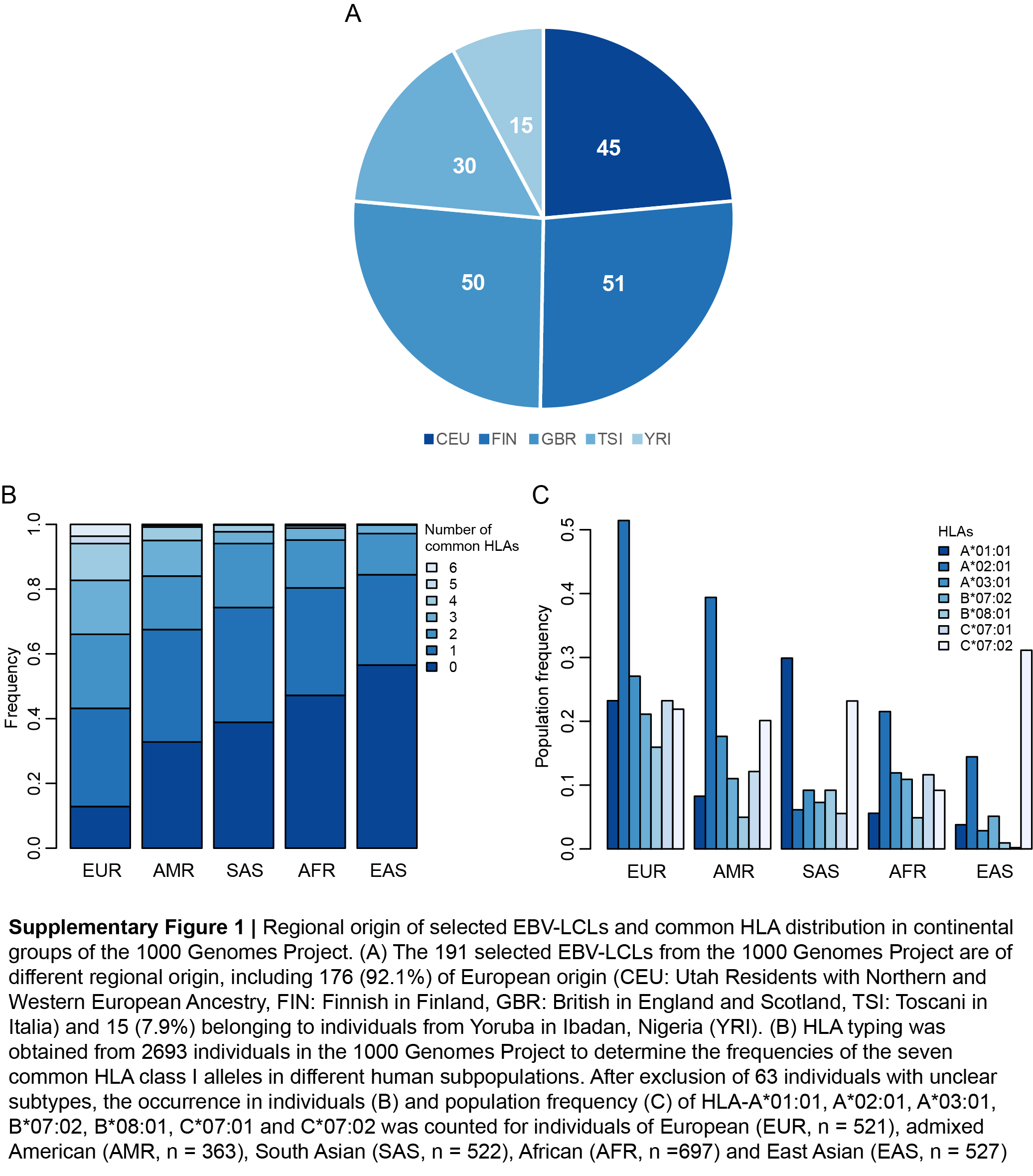

Supplement: FIGURE S1 — Regional origin of selected EBV-LCLs and common HLA distribution in continental groups of the 1000 Genomes Project. (A) The 191 selected EBV-LCLs from the 1000 Genomes Project are of different regional origin, including 176 (92.1%) of European origin (CEU: Utah Residents with Northern and Western European Ancestry, FIN: Finnish in Finland, GBR: British in England and Scotland, TSI: Toscani in Itay) and 15 (7.9%) belonging to individuals from Yoruba in Ibadan, Nigeria (YRI). (B) HLA typing was obtained from 2693 individuals in the 1000 Genomes Project to determine the frequencies of the seven common HLA class I alleles in different human subpopulations. After exclusion of 63 individuals with unclear subtypes, the occurrence in individuals (B) and population frequency (C) of HLA-A∗01:01, A∗02:01, A∗03:01, B∗07:02, B∗08:01, C∗07:01, and C∗07:02 was counted for individuals of European (EUR, n = 521), admixed American (AMR, n = 363), South Asian (SAS, n = 522), African (AFR, n = 697) and East Asian (EAS, n = 527) background. [file Image_1.jpg]
